# Ambient Air Pollutions Are Associated with Vitamin D Status

**DOI:** 10.3390/ijerph18136887

**Published:** 2021-06-27

**Authors:** Chenlu Yang, Dankang Li, Yaohua Tian, Peiyu Wang

**Affiliations:** 1 Department of Nutrition and Food Hygiene, School of Public Health, Peking University, No.38 Xueyuan Road, Beijing 100191, China; yangchenluwork@126.com; 2 Department of Maternal and Child Health, School of Public Health, Huazhong University of Science and Technology, No.13 Hangkong Road, Wuhan 430030, China; ldk199208@163.com (D.L.); yaohua_tian@hust.edu.cn (Y.T.); 3 Department of Social Medicine and Health Education, School of Public Health, Peking University, No.38 Xueyuan Road, Beijing 100191, China

**Keywords:** air pollution, vitamin D, UK cohort

## Abstract

Evidence on the effect of ambient air pollution on vitamin D is limited. This study aimed to examine the association of air pollution exposure with serum levels of 25-hydroxyvitamin D (25OHD) using UK Biobank health datasets. A total of 448,337 subjects were included in this analysis. Land Use Regression was applied to assess individual exposures to particulate matter with diameters ≤2.5 µm (PM_2.5_), ≤10 µm (PM_10_), nitrogen dioxide (NO_2_), and nitrogen oxides (NO_x_). Linear regression models evaluated the associations between air pollutants and serum vitamin D levels after adjustment of a series of confounders. All analyzed air pollutants were negatively associated with serum vitamin 25OHD levels. After adjusting for potential confounders, a 10 μg/m^3^ increase in concentrations of PM_2.5_, PM_10_, NO_x_, and NO_2_ was associated with −9.11 (95%CI: −13.25 to −4.97), −2.47 (95%CI: −4.51 to −0.43), −0.56 (95%CI: −0.82 to −0.30), and −1.64 (95%CI: −2.17 to −1.10) nmol/L decrease in serum vitamin 25OHD levels, respectively. Interaction analyses suggested that the effects of air pollution were more pronounced in females. In conclusion, long-term exposures to ambient PM_2.5_, PM_10_, NO_x_, and NO_2_ were associated with vitamin D status in a large UK cohort.

## 1. Introduction

Vitamin D plays a critical role in maintaining phosphorus and calcium homeostasis [1]. In addition, vitamin D is involved in regulating cell growth and immunity [2]. Sufficient vitamin D levels are vital to sustain human health. Vitamin D deficiency has been reported to be associated with various health outcomes, including fractures, osteoporosis, autoimmune diseases, cancers, microbial infections, diabetes, and cardiovascular disease [3,4,5]. An estimated one billion people suffer from vitamin D deficiency or insufficiency worldwide [3]. Vitamin D deficiency has been recognized as an important public health concern, and vitamin D has received growing attention.

Serum vitamin D levels are influenced by many factors, such as age, diet, and chronic diseases [6]. There are few natural dietary sources of vitamin D, and serum vitamin D is mainly synthesized in the skin upon sunlight exposure [7]. Insufficient cutaneous absorption of UVB and inadequate radiation are closely related to vitamin D deficiency [8]. Air pollution is a main determinant of the ground level of UVB. Recently, a handful of studies tried to assess the effects of air pollution on serum vitamin D levels, but the findings have been inconclusive [9,10,11,12,13]. The inconsistency might be caused by small sample sizes, and differences in air pollution metrics, geographical conditions, and exposure assessments.

In this study, we examine the relations between long-term exposures to ambient air pollutants, including particulate matter with diameters ≤2.5 µm (PM_2.5_) and ≤10 µm (PM_10_), nitrogen dioxide (NO_2_) and nitrogen oxides (NO_x_), and serum levels of 25-hydroxyvitamin D (25OHD) using UK Biobank health datasets.

## 2. Materials and Methods

### 2.1. Data Source and Study Population

The UK Biobank recruited approximately 0.5 million community-based volunteers aged 37 to 73 between 2006 and 2010 from 22 assessment centers located throughout England, Wales, and Scotland [14]. Participants provided health information through touch-screen questionnaires (e.g., demographic, health, and lifestyle information) and anthropometric measurements. All participants gave written consent, and ethical approval was obtained from the North West Multi-Centre Research Ethics Committee (London, UK). The characteristics of participants are provided on the website of UK Biobank (www.ukbiobank.ac.uk, 7 April 2021, date last accessed). In the current study, we excluded those with missing values on air pollution exposures and serum vitamin D concentrations (*n* = 54,357), leaving a total of 448,337 participants in the main analysis.

### 2.2. Assessment of Air Pollution

The UK Biobank used air pollution data obtained from the Small Area Health Statistics Unit in conjunction with the BioShaRE-EU Environmental Determinants of Health Project (https://biobank.ndph.ox.ac.uk/showcase/label.cgi?id=115, 7 April 2021, date last accessed). Annual particulate matter of up to 10 μm diameter (PM_10_ and PM_2.5_), nitrogen dioxide (NO_2_), and nitrogen oxides (NO_x_) were modeled for the year 2010 using a Land Use Regression (LUR) model developed as part of the European Study of Cohorts for Air Pollution Effects (ESCAPE; http://www.escapeproject.eu/, 7 April 2021, date last accessed). The LUR was applied to calculate the spatial variations of annual average concentrations of air pollutants according to the geographic predictor variables obtained from the Geographical information system, such as traffic variables, land use, topography, and population density [15,16]. More detailed information about the model and model performance has been described elsewhere. Leave-one-out cross-validation, where each site is left out sequentially while the included variables of the models are left unchanged, showed good model performance for PM_2.5_, PM_10_, and NO_2_ (cross-validation R^2^ = 77%, 88% and 87%, respectively) [15,16]. 

### 2.3. Measurement of Vitamin D 

Details about serum biomarker measurements and assay performances have been described in the online UK Biobank Showcase (http://biobank.ndph.ox.ac.uk/showcase/showcase/docs/serum_biochemistry.pdf, 10 April 2021, date last accessed). Briefly, serum levels of 25OHD (a proxy for vitamin D levels) measurement (in nanomole per liter) was performed by the immunoassay analyzer DiaSorin Liaison XL. The coefficients of variation for serum 25(OH)D ranged from 5.0 to 6.1%, indicating that the measurements were reliable and acceptable. Moreover, the assay of serum 25OHD was registered with an external quality assurance (EQA) scheme (RIQAS Immunoassay Speciality 1) to verify accuracy and the external quality assurance result was 100% for vitamin D.

### 2.4. Covariates

We included covariates that could potentially confound the association between exposure to air pollution and vitamin D based on current knowledge. Gender was binary coded as women and men. Ethnicity was coded as a six-factor variable: White; Mixed; Asian; Black; Chinese; and Other. Employment was coded as a three-factor variable: employed; retired; unemployed; home maker; or other (including unable to work due to illness/disability, doing unpaid or voluntary work, full or part-time student, and none of the above). Education was coded as a five-factor variable: College or university degree; O levels (includes the School Certificate), General Certificate of Secondary Educations (GCSEs), or Certificate of Secondary Educations (CSEs); A levels or AS levels (includes the Higher School Certificate); National Vocational Qualification (NVQ), Higher National Diploma (HND), Higher National Certificate (HNC), or other professional; and None. Smoking status was coded as never, previous, and current smokers, and drinking status was coded as never, previous, and current drinkers. We classified the measured BMI status into three groups: (a) <25 kg/m^2^ (normal weight); (b) 25–30 kg/m^2^ (overweight); (c) ≥30 kg/m^2^ (obesity). We also controlled for season of blood collection (spring: March–May; summer: June–August; autumn: September–November; and winter: December–February). Vitamin D supplementation usage and vitamin intake was binary coded as none or have based on a 24-h dietary recall of the previous day. Physical activity was assessed with a modified version of the International Physical Activity Questionnaire (IPAQ) that recorded total physical activity (e.g., low, moderate, and high) [17].

### 2.5. Statistical Analysis

We documented descriptive characteristics of participants with the mean (standard deviation) for continuous variables and number (percent) for categorical variables. We explored associations between air pollution and vitamin D using multivariable linear regression model. Results are reported for crude associations (unadjusted) in model 1 and then adjusted for age, sex, ethnicity, body mass index, education, employment status, smoking status, drinking status, physical activity, season of blood collection, vitamin D supplement, and vitamin intake in Model 2. In addition, we also evaluated the associations in subgroups stratified by gender, age (age <60 and age ≥60, separately), and the season of blood collection. Tests for interactions across subgroups were performed using the Wald test. The results were presented as change in Vitamin D levels with per 10 μg/m^3^ increase in air pollution concentrations. All analyses were performed using R version 4.0.2 (R Foundation for Statistical Computing, https://www.r-project.org/, 25 April 2021, date last accessed), and two-sided *p* < 0.05 were considered statistically significant.

## 3. Results

Table 1 shows the characteristics of the study participants and distribution of air pollutants. This analysis included 448,337 individuals with a mean age of 56.47 (SD, 8.12) years, and 53.6% were women. The mean (SD) values of annual concentrations of PM_2.5_, PM_10_, NO_x_, and NO_2_ were 9.99 (1.06), 16.23 (1.90), 44.02 (15.48), and 26.65 (7.56) μg/m^3^, respectively.

Table 2 presents the associations of air pollutants with serum vitamin D levels. After adjustment for potential confounders, a 10 μg/m^3^ increase in concentrations of PM_2.5_, PM_10_, NO_x_, and NO_2_ was associated with a −9.11 (95%CI: −13.25 to −4.97), −2.47 (95%CI: −4.51 to −0.43), −0.56 (95%CI: −0.82 to −0.30), and −1.64 (95%CI: −2.17 to −1.10) nmol/L decrease in serum vitamin 25OHD levels, respectively.

The association between air pollution and serum vitamin D was more evident in females (Table 3). Each 10 μg/m^3^ increase PM_2.5_, PM_10_, NO_x_, and NO_2_ concentrations was related to a −14.69 (95%CI: −20.70 to −8.69), −4.25 (95%CI: −7.20 to −1.30), −0.69 (95%CI: −1.06 to −0.32), and −1.94 (95%CI: −2.71 to −1.17) nmol/L decrease in serum vitamin 25OHD levels, respectively. We found no evidence for an effect modification by age or the season of blood collection (Table 4 and Table 5).

All models were adjusted for age, ethnicity, body mass index, education, employment status, smoking status, drinking status, physical activity, vitamin D supplement, and vitamin intake.

## 4. Discussion

To our knowledge, this is the largest analysis to date to evaluate the association between ambient air pollution and serum vitamin D. In this nationwide cohort, we observed that long-term exposures to PM_2.5_, PM_10_, NO_x_, and NO_2_ were associated with decreased serum 25OHD levels. These associations remained after extensive controlling of personal characteristics and lifestyle factors. We further found that the associations were more evident in females.

Several studies have assessed the effects of air pollution on vitamin D in infants, children, young adults, pregnant women, and the general population [9,10,12,18,19,20]. For example, Baïz et al. [11] demonstrated that gestational exposures to ambient urban levels of NO_2_ and PM_10_ were associated with a decrease in the 25OHD cord blood serum level in 375 mother-child pairs. Similarly, two studies reported positive association between air pollution exposure and vitamin D deficiency in India [18] and Iran [19]. In addition, two studies suggested that prenatal exposure to particulate air pollution may play an independent role in maternal vitamin D deficiency [9,12]. Recently, a cross-sectional population-based study in China demonstrated that vitamin D deficiency was related to air pollution [10].

Our findings are consistent with prior studies indicating that subjects exposed to higher air pollution levels have lower vitamin D levels or are at increased risk of vitamin D deficiency. Nevertheless, previous studies generally used citywide monitoring measurements as a proxy of air pollution, instead of assessing individual exposure to air pollution. For example, Manicourt et al. used the mean of values recorded by official agencies as exposure, and found that postmenopausal women exposed to higher ozone pollution had higher prevalence of vitamin D deficiency [21]. In this study, we applied Land Use Regression to assess individual annual average exposure, taking into account land use, traffic variables, topography, and population density.

The mechanism underlying the link between air pollution and vitamin D remain unclear. Under the influence of UVB, skin synthesis of vitamin D accounts for about 90% of vitamin D production in humans. Studies have reported that ambient air pollutants can absorb and diffuse solar irradiation, thus decreasing ground levels of UVB [22]. Therefore, we hypothesized that air pollution decreased serum vitamin D levels mainly through decreasing the ground levels of UVB. In addition, people in areas with high air pollution levels generally spend less time outdoors. All these factors act synergistically to decrease the availability of vitamin D.

Although the decrease in serum vitamin D associated with air pollution was small, air pollution is ubiquitous in this population. Vitamin D deficiency or insufficiency affects approximately one billion people worldwide [3], and is closely related to the vast majority of health conditions [3,4,5]. Considering the high prevalence of vitamin D deficiency and its associated health impacts, our findings about air pollution-related effects on vitamin D have great implications to enhance primary prevention efforts aimed at reducing vitamin D deficiency from a public health perspective.

Our study was based on a national cohort with good quality control, ensuring good statistical power and generalizability of our findings. Additionally, a series of socioeconomic and lifestyle factors were adjusted in the model, decreasing potential confounding effects. Our findings contributed to the limited evidence about the effects of air pollution on serum vitamin D.

Our analysis also has some limitations. First, air pollution exposures were assessed at the place of residence, resulting in exposure misclassification. This misclassification is generally non-differential and tends to bias the risk estimates downward [23]. Second, the cross-sectional study design limited the ability to examine temporal patterns. Longitudinal studies are needed to confirm causal associations of air pollution with vitamin D. Third, the 4-year period in this study is not a long time to assess the risk. However, the significant association between air pollution and vitamin D was still observed, suggesting that the effect of air pollution on vitamin D status was considerable even over a few years. Finally, although many potential confounders were controlled in the model, we cannot exclude potential residual confounding from unmeasured variables, e.g., temperature.

## 5. Conclusions

Our data indicated that long-term exposure to ambient PM_2.5_, PM_10_, NO_x_, and NO_2_ was associated with vitamin D status in a large UK cohort.

## Figures and Tables

**Table 1:** Characteristics of the participants included (N = 448,337).

Characteristics	Mean (SD)/Number (%)
N	448,337
Age (mean (SD))	56.47 (8.12)
Ethnicity (%)	
White	423,151 (94.8)
Mixed	2632 (0.6)
Asian	8031 (1.8)
Black	7064 (1.6)
Chinese	1405 (0.3)
Other	3961 (0.9)
Gender (%)	
Women	240,119 (53.6)
Men	208,218 (46.4)
Education (%)	
None	75,938 (17.1)
College or university degree	145,638 (32.9)
O levels, GCSEs, or CSEs	94,654 (21.4)
A levels or AS levels	49,848 (11.3)
NVQ, HND, HNC, or other professional	76,993 (17.4)
Employment (%)	
Employed	258,576 (58.0)
Retired	147,871 (33.1)
Unemployed, home maker, or other	39,643 (8.9)
BMI categories (%)	
Normal weight	148,602 (33.3)
Overweight	189,312 (42.4)
Obesity	108,672 (24.3)
Smoke (%)	
Never	244,233 (54.7)
Previous	155,172 (34.8)
Current	46,688 (10.5)
Drink (%)	
Never	19,295 (4.3)
Previous	16,066 (3.6)
Current	411,883 (92.1)
Physical activity (%)	
Low	68,074 (18.8)
Moderate	147,763 (40.7)
High	146,972 (40.5)
Season of blood collection (%)	
Spring	41,175 (24.2)
Summer	49,605 (29.1)
Autumn	44,113 (25.9)
Winter	35,340 (20.8)
Vitamin D supplement (%)	
none	437,441 (98.2)
have	8149 (1.8)
Vitamin intake (%)	
none	37,716 (59.2)
have	26,034 (40.8)
Air pollution	
PM_2.5_, μg/m^3^	9.99 (1.06)
PM_10_, μg/m^3^	16.23 (1.90)
NO_x_, μg/m^3^	44.02 (15.48)
NO_2_, μg/m^3^	26.65 (7.56)

Abbreviations: N, number; SD, standard deviation; GCSEs, general certificate of secondary educations; CSEs, certificate of secondary educations; NVQ, national vocational qualification; HND, higher national diploma; HNC, higher national certificate; PM, particulate matter; NO_2_, nitrogen dioxides; NO_x_, nitrogen oxides.

**Table 2:** Associations of air pollution and Vitamin D.

Air Pollution	Model 1		Model 2	
	β-Value (95%CI)	*p*-Value	β-Value (95%CI)	*p*-Value
PM_2.5_	−19.76 (−20.37, −19.15)	<0.001	−9.11 (−13.25, −4.97)	<0.001
PM_10_	−5.19 (−5.53, −4.85)	<0.001	−2.47 (−4.51, −0.43)	0.018
NO_x_	−1.39 (−1.43, −1.35)	<0.001	−0.56 (−0.82, −0.30)	<0.001
NO_2_	−3.35 (−3.43, −3.27)	<0.001	−1.64 (−2.17, −1.10)	<0.001

Abbreviations: PM, particulate matter; NO_2_, nitrogen dioxides; NO_x_, nitrogen oxides; CI, confidence interval. Associations are measured per 10 μg/m^3^ change for PM_2.5_, PM_10_, NO_x_, and NO_2_ level. β-value indicates the value of decrease in vitamin D levels associated with a unit increase of air pollutant exposure. Model 1, crude; Model 2, adjusted for age, sex, ethnicity, body mass index, education, employment status, smoking status, drinking status, physical activity, season of blood collection, vitamin D supplement, and vitamin intake.

**Table 3:** Associations between air pollution (per 10 μg/m^3^ increase) and vitamin D in subgroups stratified by gender.

Air Pollutions	Women		Man		*p*-Value for Interaction
	*β*-Value (95%CI)	*p*-Value	*β*-Value (95%CI)	*p*-Value	
PM_2.5_	−14.69 (−20.70, −8.69)	<0.001	−2.75 (−8.41, 2.92)	0.342	0.006
PM_10_	−4.25 (−7.20, −1.30)	<0.001	−0.49 (−3.28, 2.31)	0.733	0.080
NO_x_	−0.69 (−1.06, −0.32)	<0.001	−0.39 (−0.74, −0.03)	0.032	0.271
NO_2_	−1.94 (−2.71, −1.17)	<0.001	−1.25 (−2.00, −0.51)	<0.001	0.200

Abbreviations: PM, particulate matter; NO_2_, nitrogen dioxides; NO_x_, nitrogen oxides; CI, confidence interval. *p*−value for the interaction between air pollution and gender. β-value indicates the value of decrease in vitamin D levels associated with a unit increase of air pollutant exposure. All models were adjusted for age, ethnicity, body mass index, education, employment status, smoking status, drinking status, physical activity, season of blood collection, vitamin D supplement, and vitamin intake.

**Table 4:** Associations between air pollution (per 10 μg/m^3^ increase) and vitamin D in subgroups stratified by age.

Air Pollutions	Age <60		Age ≥60		*p*-Value for Interaction
	*β*-Value (95%CI)	*p*-Value	*β*-Value (95%CI)	*p*-Value	
PM_2.5_	−8.95 (−14.33, −3.56)	0.001	−9.87 (−16.37, −3.36)	0.003	0.791
PM_10_	−3.34 (−5.97, −0.71)	0.013	−1.38 (−4.62, −1.86)	0.404	0.954
NO_x_	−0.56 (−0.91, −0.22)	0.002	−0.58 (−0.97, −0.20)	0.003	0.359
NO_2_	−1.71 (−2.41, −1.01)	<0.001	−1.62 (−2.45, −0.78)	<0.001	0.487

Abbreviations: PM, particulate matter; NO_2_, nitrogen dioxides; NOx, nitrogen oxides; CI, confidence interval. *p*-value for the interaction between air pollution and age group. β-value indicates the value of decrease in vitamin D levels associated with a unit increase of air pollutant exposure. All models were adjusted for gender, sex, ethnicity, body mass index, education, employment status, smoking status, drinking status, physical activity, season of blood collection, vitamin D supplement, and vitamin intake.

**Table 5:** Associations between air pollution (per 10 μg/m^3^ increase) and vitamin D in subgroups stratified by season of blood collection.

	Spring		Summer		Autumn		Winter		*p*-Value for Interaction
	β-Value (95%CI)	*p*-Value	β-Value (95%CI)	*p*-Value	β-Value (95%CI)	*p*-Value	β-Value (95%CI)	*p*-Value	
PM_2.5_	−17.67 (−27.53, −7.80)	<0.001	−3.95 (−10.60, 2.69)	0.243	−11.03 (−19.02, −3.04)	0.007	−5.09 (−15.47, 5.28)	0.336	0.808
PM_10_	−4.88 (−9.43, −0.33)	0.036	−0.43 (−3.70, 2.84)	0.795	−3.21 (−7.19, 0.77)	0.114	−2.53 (−8.04, 2.98)	0.367	0.789
NO_x_	−1.13 (−1.75, −0.52)	<0.001	−0.35 (−0.75, 0.06)	0.095	−0.68 (−1.18, −0.18)	0.008	−0.07 (−0.72, 0.58)	0.833	0.408
NO_2_	−2.29 (−3.58, −1.00)	<0.001	−1.46 (−2.31, −0.61)	<0.001	−1.90 (−2.92, −0.88)	<0.001	−0.40 (−1.79, 0.98)	0.568	0.173

Abbreviations: PM, particulate matter; NO_2_, nitrogen dioxides; NO_x_, nitrogen oxides; CI, confidence interval. *p*-value for the interaction between air pollution and gender. β-value indicates the value of decrease in vitamin D levels associated with a unit increase of air pollutant exposure.

## Data Availability

Data supporting reported results can be found from the website of UK Biobank (www.ukbiobank.ac.uk, 1 May 2021, date last accessed) upon application.
